# Miniaturized Embryo Array for Automated Trapping, Immobilization and Microperfusion of Zebrafish Embryos

**DOI:** 10.1371/journal.pone.0036630

**Published:** 2012-05-14

**Authors:** Jin Akagi, Khashayar Khoshmanesh, Barbara Evans, Chris J. Hall, Kathryn E. Crosier, Jonathan M. Cooper, Philip S. Crosier, Donald Wlodkowic

**Affiliations:** 1 The BioMEMS Research Group, School of Chemical Sciences, University of Auckland, Auckland, New Zealand; 2 School of Electrical and Computer Engineering, RMIT University, Melbourne, Australia; 3 Department of Molecular Medicine and Pathology, School of Medical Sciences, University of Auckland, Auckland, New Zealand; 4 School of Engineering, University of Glasgow, Glasgow, United Kingdom; 5 School of Applied Sciences, RMIT University, Melbourne, Australia; Istituto Dermopatico dell’Immacolata, Italy

## Abstract

Zebrafish (*Danio rerio*) has recently emerged as a powerful experimental model in drug discovery and environmental toxicology. Drug discovery screens performed on zebrafish embryos mirror with a high level of accuracy the tests usually performed on mammalian animal models, and fish embryo toxicity assay (FET) is one of the most promising alternative approaches to acute ecotoxicity testing with adult fish. Notwithstanding this, automated *in-situ* analysis of zebrafish embryos is still deeply in its infancy. This is mostly due to the inherent limitations of conventional techniques and the fact that metazoan organisms are not easily susceptible to laboratory automation. In this work, we describe the development of an innovative miniaturized chip-based device for the *in-situ* analysis of zebrafish embryos. We present evidence that automatic, hydrodynamic positioning, trapping and long-term immobilization of single embryos inside the microfluidic chips can be combined with time-lapse imaging to provide real-time developmental analysis. Our platform, fabricated using biocompatible polymer molding technology, enables rapid trapping of embryos in low shear stress zones, uniform drug microperfusion and high-resolution imaging without the need of manual embryo handling at various developmental stages. The device provides a highly controllable fluidic microenvironment and post-analysis eleuthero-embryo stage recovery. Throughout the incubation, the position of individual embryos is registered. Importantly, we also for first time show that microfluidic embryo array technology can be effectively used for the analysis of anti-angiogenic compounds using transgenic zebrafish line (fli1a:EGFP). The work provides a new rationale for rapid and automated manipulation and analysis of developing zebrafish embryos at a large scale.

## Introduction

Small model organisms offer advantages over cell lines and isolated tissues by providing analysis under normal physiological milieu of the whole organism [1]–[3]. This opens up enhanced analytical capabilities that cannot be easily replicated *in vitro* using isolated primary cells, cell lines and/or tissue cultures [1]–[3]. In this context, zebrafish (*Danio rerio*) is gaining considerable interest as a convenient experimental model. Small size, optical transparency of organs and simple husbandry make *D.rerio* embryos, eleuthero-embryos and juvenile stages as the ideal models for large scale pharmacological and toxicological studies [2], [3]. Abundant experimental techniques and molecular tools have facilitated the use of zebrafish as the model of choice for studies of many human diseases [3]. It has already been reported as a powerful and versatile vertebrate system that can facilitate accelerated drug discovery [3]–[5]. Furthermore, fish embryo toxicity assay (FET) is one of the most promising alternative approaches to classical ecotoxicity testing with adult fish, already standardized at the international level according to OECD and US EPA guidelines [6]–[8].

Paradoxically, analysis of small model organisms such as fish embryos and juveniles in a high-throughput and high-content manner is still a challenging task [1], [2], [9]. This is mostly due to the inherent limitations of conventional techniques and the fact that metazoan organisms are not easily susceptible to laboratory automation. Despite some emerging progress in cytometric large particle analysis, the embryo handling and treatment are mainly performed manually and bioassays carried out under sub-optimal, static microtiter plate conditions [1], [7], [9]–[11].

Not surprisingly, implementation of miniaturized chip-based systems for *in-situ* analysis of small model organisms is attracting a rapidly growing interest [1], [12]. Mushrooming reports have recently showed automated manipulation and immobilization of micron-sized organisms such as *C.elegans and D.melanogaster* [1], [12]–[15]. Development of chip-based devices able to automatically manipulate millimeter and sub-millimeter scale organisms such as fish and amphibian embryos is, however, still in its infancy but can prospectively yield new avenues for drug discovery and ecotoxicity screening [1]. Several attempts have been recently made and involved (i) embryo culture in segmented flow inside the PTFE tubes [16]; (ii) use of electrowetting-on-dielectric (EWOD) technique for transport of live zebrafish embryos in small droplets [17]; (iii) the use of a glass microwell flow-through system [18]; (iv) the development of a polymeric microwell array with integrated gradient generator [19]; and finally (v) vertebrate automated screening technology (VAST) for automated manipulation and imaging of several days old larvae in a glass microcapillary mounted on a 3D robotic manifold [20]. These approaches have recently been reviewed [1] and none of them allow for a high-speed, automated and gentle hydrodynamic positioning, trapping and long-term immobilization of large numbers of single vertebrate embryos for real-time developmental analysis [1].

In this work, we for the first time describe an innovative miniaturized embryo array that registers each embryo throughout the analysis in a single addressed location, provides highly controllable fluidic microenvironment and post-analysis specimen recovery. In contrast to any previously described zebrafish chips, it allows for a one-step automatic loading, hydrodynamic positioning, trapping and long-term immobilization of single embryos inside the microfluidic chips. The miniaturized devices were fabricated using a high-speed laser fabrication system adequate for both rapid prototyping and medium scale production. Importantly, we also for first time show that microfluidic embryo array technology can be effectively used for the analysis of anti-angiogenic compounds using transgenic zebrafish line (fli1a:EGFP) [21]. The work provides a new rationale for rapid and automated manipulation and analysis of developing transgenic zebrafish embryos using microfluidic devices at a large scale.

## Materials and Methods

### Chip Design and Fabrication

**Figure 1:**
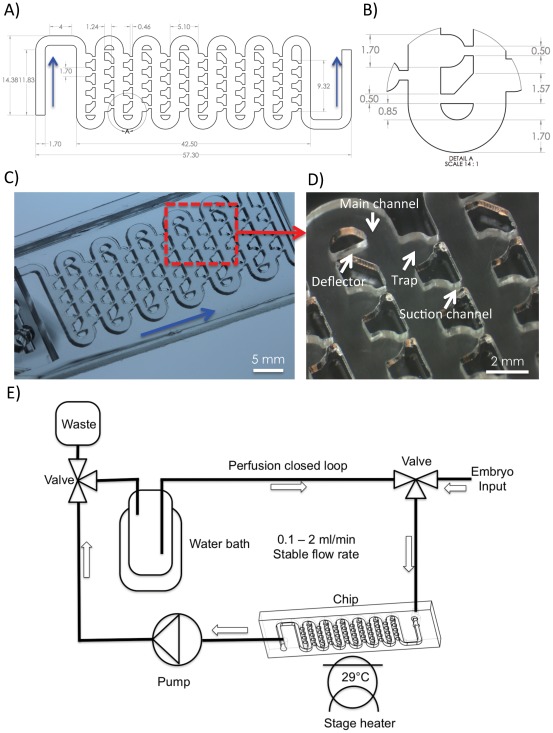
Microfluidic embryo array chip for automatic trapping and immobilization of zebrafish embryos: **A**) CAD drawing showing the design of the serpentine embryo array device; **B**) Magnified detail section with the hydrodynamic deflector, embryo trap, and a suction channel; **C**) Photograph showing the assembled device moulded in the elastomer PDMS and bonded to the glass slide; **D**) Microphotograph showing a section with six embryo traps; **E**) Schematic showing the off-chip interconnections and hardware components actuating the microfluidic device. For details refer to the Materials and Methods. Blue arrows depict the direction of fluid flow direction.

The microfluidic chip was designed and modeled using CorelDraw X4 (Corel Corporation, Ontario, Canada) and SolidWorks 2011 (Dassault Systemes SolidWorks Corp, Concord, MA, USA) CAD packages. Subsequent prototyping and fabrication was performed using a non-contact, 30 W CO_2_ laser cutting system equipped with a High Power Density Focusing Optics (HPDFO)™ (Universal Laser Systems, Scottsdale, AZ, USA). The master shape was laser cut in 1.5 mm thick poly-methyl methacrylate sheet (PMMA/Acrylic; PSP Plastics Ltd, Auckland, New Zealand) to form the integrated channel and trapping region structure (Figure 1). The master was then thermally bonded to a 25**×**75**×**2 mm PMMA sheet at 110°C for up to 2 hours in a fan assisted oven. A uniform mechanical force was applied using two mini C-clamps to provide equal force distribution during PMMA layer bonding. The bonded PMMA master was then used for replica molding in poly(dimethylsiloxane) (PDMS; Sylgard 184; DowCorning Corp, Midland, MI, USA), as described earlier [22]. Briefly, the PDMS was mixed at a 10∶1 (w/w) ratio of elastomer base to curing agent and degassed at 40 Torr to remove any residual air bubbles. PDMS was then poured on a PMMA master to achieve approximately 5 mm thickness and cured thermally at 80°C for up to 1 hour. Cured PDMS devices were mechanically diced and bonded to the glass microscope slides using oxygen plasma surface activation. Tubing interconects were manually bored using an appropriate stainless steel punch hole, as described earlier [22], [23].

### Computational Analysis and Multiphysics Modeling

In order to estimate the velocities throughout the flow domain, shear stress over the chorion surface, diffusion of the drug/dye and the immobilization force that holds embryos inside the traps, two-dimensional (2D) and three-dimensional (3D) models of the device were created with virtual embryos as spherical structures inside the traps. The simulation was performed using Gambit 2.3 software (Fluent, Lebanon, NH, USA) to create the geometry and mesh generation. Finite-volume based Fluent 6.3 software (Fluent, Lebanon, NH, USA) was subsequently used to solve the associated differential equations governing the balance of mass, momentum, chemical species, as given below:
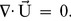
(1)

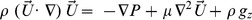
(2)

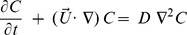
(3)where, 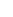
and P are the velocity vector and the pressure of the flow, *C* is the concentration of species, *ρ*, µ and *D* are the density, dynamic viscosity and diffusion coefficient of chemical species, *t* is time, and *g_z_* is the gravitational acceleration.

Boundary conditions consisted of a flow rate between 100–2000 µl/min at the inlet and a pressure set to 0 Pa at the outlet. The other surfaces including the bottom, top and sidewalls of the channels as well as the embryo chorions were set to no-slip condition. The highest Reynolds number obtained at the maximum flow rate is 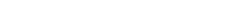 indicating the laminar characteristics of our miniaturized system.

### Chip Setup, Loading and Operation

The device was directly connected to the external high-precision Miniplus Evolution peristaltic pump equipped with a MF1 pump head (Gilson Inc, Middleton, WI, USA) using 1/16″ polyurethane tubing (Cole-Parmer Instrument Company, Vernon Hills, Illinois, USA) with an internal diameter allowing for the free passage of zebrafish embryos. The PVC calibrated tubing (1.02 mm ID; Gilson Inc) was mounted inside the pump to provide flow rates over the desired range (100–2000 µl/min). Devices were primed with 70% ethanol (v/v) to help wet the PDMS and reduce the nucleation and persistence of air bubbles. Chips then were filled with standard E3 fish medium. The chips were positioned on a microscope stage and embryos with intact chorion between 6–24 hours post-fertilization (hpf) were loaded by aspirating single embryos one by one at the flow rates of up to 2 ml/min. The pump was adjusted to provide a continuous negative pressure (nominal withdrawal mode at up to 2 ml/min). Embryo loading and trapping was confirmed microscopically. After loading, the chips were placed on a microprocessor-controlled heating stage (IKA Works, Petaling Jaya, Malaysia) and connected to a 250 ml glass reservoir (Schott AG, Mainz, Germany) mounted inside a waterbath (Julabo Labortechnik GmbH, Seelbach, Germany). Heated medium was recirculated in a closed loop perfusion for up to 72 hours allowing for an optimal embryo development temperature of about 28.5±0.5°C inside the PDMS chip.

### Zebrafish Husbandry

Adult zebrafish were kept in a 14 hour light, 10 hour dark cycle fish facility and fed twice daily with artemia and once daily with dry feed. Wild-type zebrafish (*Danio rerio* (AB line; Zebrafish International Resource Center, Oregon, Eugene, OR, USA) Animal research was conducted with approval from The University of Auckland Animal Ethics Committee (approval ID R661/1) [24].

### Embryo Culture, Treatment and Phenotype Analysis

Zebrafish embryos were obtained from random pair-wise mating and natural spawning. Embryos were then collected in embryo medium E3 and rinsed to remove any debris and dead embryos. Embryos were kept at 28.5±0.5°C in E3 medium and developmentally staged as described earlier [24], [25]. The embryos of 4–6 and/or 24 hpf were selected for experiments. Various parameters were analyzed according to: (i) Lethal endpoints (cumulative mortality): coagulation, tail detachment, lack of somite formation; (ii) Sublethal developmental endpoints: development of eyes, spontaneous movement, heartbeat and blood circulation, pigmentation, formation of edemata; (iii) Endpoints of teratogenicity: malformation of the head, malformation of tail, yolk deformation general growth retardation [6], [7], [26]. Hatchling potential and time was also evaluated [6], [7]. Following all experiments, the hatched eleuthero-embryos were euthanized at −20°C. For the mass transfer experiments where preservation of viability was not important the chips were perfused with 0.04% Trypan Blue dye (Life Technologies Corp, CA, USA) in tap water.

### Zebrafish in vivo Angiogenesis Assay

Friend leukaemia integration 1a transgenic zebrafish line (fli1a:EGFP)^y1^ expressing enhanced green fluorescent protein (EGFP) in the vasculature throughout the development of zebrafish embryos was used [21]. The 4 hpf (hours post-fertilization) embryos were collected and placed in 5 ml of E3 medium supplemented with penicillin/streptomycin (Life Technologies Corp) and maintained in the dark overnight at the 22°C to slow down the process of embryogenesis. Subsequently all non-fertilized embryos were removed and embryos at 16 hpf stages were loaded on each chip. Up to 20 embryos per chip were loaded using E3 medium as a carrier. Following embryo docking and immobilization the E3 medium in each chip was replaced with the E3 solution supplemented with either 1 µM of selective VEGFR inhibitor AV951 (Tivozanib; AVEO Pharmaceuticals Inc, Cambridge, MA, USA) or vehicle (DMSO, Life Technologies Corp) [27], [28]. The medium was also supplemented with 0.003% 1-phenyl-2-thiourea (PTU, Life Technologies Corp) to maintain the optical transparency of the zebrafish embryos. Chips were then positioned on a heating stage at 28–29°C and continuous closed-loop perfusion at the flow rate of 100 µl/min was maintained for the 48 hours. The recirculated medium was heated to the 28–29°C using miniaturized water bath. A 0.2 mg/ml of Tricaine mesylate buffered solution was perfused through the chips 15 minutes before image acquisition. This was maintained only for the duration of imaging procedures to provide temporary anaesthesia and inhibit the intrinsic embryo movements during the fluorescent imaging. A Nikon SMZ1500 fluorescent stereomicroscope equipped with a DS-U2/L2 camera and standard FITC/GFP filter cube was used to acquire brightfield and fluorescence images of developing embryos. Intersegmental vessels (ISV) were analyzed and counted manually [21]. The complete inhibition was considered when ISV had extended to the level of the dorsal longitudinal anastomotic vessel.

### Imaging

Time-lapse imaging of developing embryos cultured on chip-based devices was performed using the Leica MZ7.5 stereomicroscope equipped with a Leica DFC295 CMOS camera and running under the LAS Multitime software (Leica Microsystems, Wetzlar, Germany). A Nikon SMZ1500 fluorescent stereomicroscope equipped with a DS-U2/L2 camera and standard FITC/GFP filter cube was used to acquire brightfield and fluorescence images of developing transgenic fli1a:EGFP embryos. Embryo trapping efficiency and mass transfer experiments were acquired using the Canon 600D Digital SLR (Canon Inc, Tokyo, Japan) equipped with a true 1∶1 macro lens (Canon EF 100 mm f/2.8 Macro Lens; Canon Inc).

### Data Analysis and Controls

Data analysis and presentation was performed using the LAS (Leica Microsystems); ImageJ (freely available at http://rsb.info.nih.gov/ij/web page); GraphPad Prism (GraphPad Software, Inc. CA, USA); SolidWorks 2011 (Dassault Systemes SolidWorks Corp) and Fluent 6.3 (Fluent) software. The Student’s t-test was applied for comparison between groups using MS Excel (Microsoft, USA) and GraphPad Prism (GraphPad Software) with significance set at p<0.05.

All control measurements are provided in detail in the Figure legends, where appropriate, but in general involved making direct comparisons between the chip-based devices and static 24-well microtiter plates or 60 mm Petri dishes (Nalge Nunc Inc, NY, USA).

## Results

### Design Rationale for Hydrodynamic Embryo Trapping

There is a noticeable lack of technologies for automated positioning, trapping and long-term immobilization of large numbers of single zebrafish embryos that can be interfaced with time-lapse imaging, video-microscopy and most importantly register position of each embryo throughout the analysis in a single location. The major obstacle against manipulation and arraying of millimeter-sized embryos in perfusion chip-based devices is linked to their substantial mass (850–1050 µg for zebrafish embryos), which leads to rapid gravitational-induced sedimentation and high momentums of translational and rotational movements. Our device was designed to overcome the challenges of large particle manipulation on Lab-on-a-Chip systems. The design was based on a 2D (one-layer), optically transparent system fabricated in a biologically compatible PDMS (Figure 1). The chip consisted of three integrated modules: (i) the main twisted shaped channel for embryo loading and medium perfusion, (ii) an array of 48 embryo traps in 12 consecutive rows, with hydrodynamic deflectors for enhanced embryo positioning, (iii) an array of small suction channels that connect traps with the main channel and provide direct hydrodynamic force to drag the embryos into the traps (Figure 1). The internal volume of the device was approximately 825.9 µl while the volume of a single trap was 2.77 µl. The advantage of the design is that it can be actuated under negative pressure with the output port connected to a microprocessor-controlled peristaltic pump and re-configurable external plumbing to open or closed-loop perfusion circuits (Figure 1). In the closed loop perfusion the device can be actuated from the reservoir’s volume of 1–2 ml. The perfusion fluidic lines can also be shortened and the valve can be configured to use tubing of very small diameter just for the drug perfusion. We calculated that the total volume of the fluid system can be reduced down to approximately 3 ml when operated in a closed loop-perfusion.

**Figure 2:**
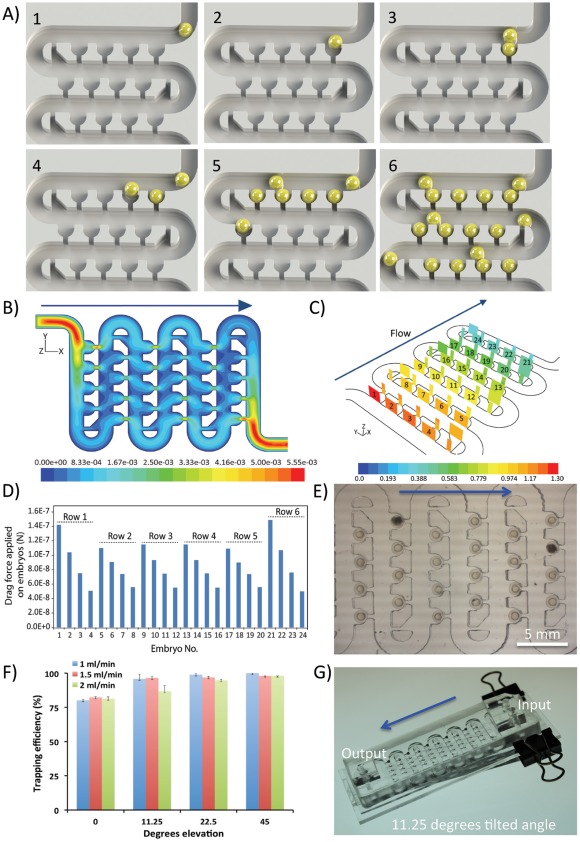
Principles and validation of embryo trapping performance: **A**) A 3D cartoon showing the embryo trapping principles: 1-embryo is aspirated from the storage vessel and injected into the main channel, 2-hydrodynamic forces guide the embryo into the trap, 3–4-next embryo is introduced and rolls on the previous one towards the next available trap, 5–6-the process is repeated till all the traps are filled with embryos, while the hydrodynamic forces keep embryos securely docked for the duration of experiments; **B**) Velocity contours (m/s) across the device at the vertical middle plane (0.75 mm from the bottom of the channel) when perfused at a flow rate of 0.4 ml/min. Due to the computational limitations only first six row were simulated; **C**) The pressure drop (Pa) across the traps when perfused at a flow rate of 0.4 ml/min; **D**) Analysis of the drag force (N) applied on embryos when device is perfused at a flow rate of 0.4 ml/min; **E**) Microphotograph showing a six row section of the device completely filled with zebrafish embryos, when perfused at a flow rate of 0.4 ml/min according to the simulations above; **F**) Experimental validation of embryo trapping efficiency at varying volumetric flow rates and tilt angles of the device; **G**) Photographs of a device mounted 11.25 degrees tilted angle stage used to perform trapping efficiency experiments as denoted in F). Blue arrows depict the direction of fluid flow and embryo movement along the serpentine channel.

The chip was designed to allow for automatic and passive trapping of individual embryos using only hydrodynamic forces (Figure 2A–C, Movie S1). For this purpose, the embryos were loaded on a chip one-by-one in approximately five-second intervals using a flexible 1.5 mm ID suction tube connected to a storage vessel. After entering the device, the embryos rolled on the bottom surface of the main channel under the influence of drag force. The dimensions of the channel allowed for a free passage of embryos traveling only in a single file (Figure 2A, Movie S1). Embryos approaching the empty traps were affected by the cross flow passing through the suction channels that changed their trajectory directly towards the traps (Figure 2A–C, Movie S1). The transverse displacement of the rows between consequent rows with the magnitude of half of the distance between two neighboring traps, generated streamline profiles enhancing the rapid docking of embryos inside the traps (Figure 2B). The embryos experienced hydrodynamic drag forces ranging from 1.5E–07 to 4.0E–08 N when perfused at the volumetric flow rate of 0.4 ml/min (Figure 2D, Figure S1). Importantly, the size and shape of the traps were designed to assure: (i) single embryo occupancy, and (ii) unobstructed passage of other embryos in the main channel following docking. Moreover, specially designed hydrodynamic deflectors at the end of each row considerably enhanced the change of particle trajectory directly towards the traps (Figure 1 and 2A–B). Interestingly, the flow velocity was highest across the first trap of each row (Figure 2B, Figure S1). This phenomenon reinforced the trapping effect, as the serpentine shape of the device resulted in an increased velocity of the embryos (1.5–2 times higher) at the turn sections of the main channel.

Subsequent embryos introduced into the device rolled freely in the main channel towards the next available traps (Movie S1). The process was repeated until all traps were occupied. Hydrodynamic forces alone were sufficient to achieve over 80% trapping efficiency when device was placed on horizontal microscope stage, confirming the computational assumptions (Figure 2E–F, Figure S1) [29]. When the device was placed on an elevated stage (tilt angle ranging from 11.25–45 degrees) the combined hydrodynamic and gravitational pull obtained nearly 100% trapping efficiency irrespectively of the flow rates applied (Figure 2F–G, Figure S1). Over 98%±2.5 of trapped embryos retained their position during the course of even long-term experiments (ca. 72 hours). Manipulation and complete tilting of the device during the perfusion process did not lead to dislodgment of any embryos. Importantly, the one-step loading and trapping process was straightforward and did not require any active on-chip or off-chip actuators apart from a single pump. After loading, the device could be disconnected by closing both input and output valves. In this scenario, however, embryos could only be held in traps by gravitational forces when the chip was tilted (Figure S1). This feature allowed for transport and/or reconnection to different hardware during the course of experiments.

The design achieved one-embryo-in-one-trap for convenient address designation and encoding to each embryo. As such it greatly facilitates: (i) staining or treatment without displacing the embryos; (ii) highly controllable fluidic microenvironment for analysis under continuous perfusion; (iii) spatial segregation of developing embryos to avoid embryo-to-embryo interaction; (iv) future applicability of customized image and data analysis software, allowing simple geometric designation of each embryo. None of these features can be achieved by using simple Petri dish approach.

### Single Embryo Microperfusion

**Figure 3:**
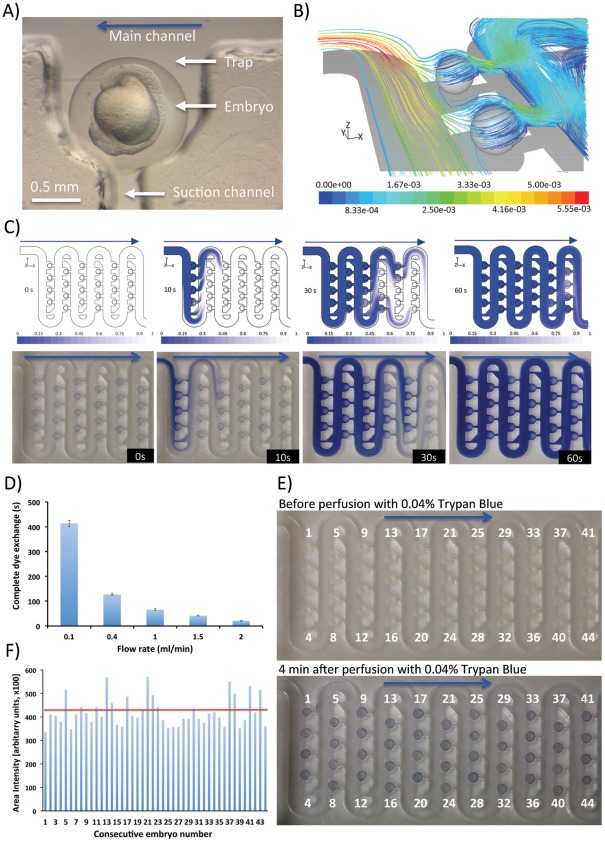
Validation of embryo microperfusion and drug delivery inside the chip: **A**) Microphotograph showing a single immobilized zebrafish embryo (circa 16 hpf); **B**) 3D streamlines of fluid around the embryos colored by flow velocity (m/s) obtained by computational fluid dynamic simulations as the fluid enters two traps occupied by docked embryos. Perfusion was simulated at a volumetric flow rate of 0.4 ml/min; **C**) Mass transfer across the simulated (upper panel) and real-world (lower panel) microfluidic array fully loaded with zebrafish embryos. Chip was perfused with a 0.04% Trypan Blue dye at a volumetric flow rate of 0.4 ml/min; **D**) Experimental analysis of the time needed for a complete dye exchange across the whole device (12 rows) at varying flow rates 0.1–2 ml/min.; **E**) Validation of the dye delivery to each embryo across the microfluidic array. Chip was perfused with a 0.04% Trypan Blue dye at a volumetric flow rate of 0.4 ml/min for up to 4 minutes. Trypan Blue was then replaced with medium; **F**) Intensity of dye across each embryo obtained by image analysis of the experiments in E). Red line denotes the average staining intensity. Blue arrows depict the direction of fluid flow and embryo movement along the serpentine channel.

Efficient exchange of circulating medium and also the uniform delivery of drugs and dyes to immobilized zebrafish embryos is an important consideration for long-term microperfusion studies in ecotoxicology and drug discovery. We observed that after docking, hydrodynamic forces pulled the embryos towards the small suction channels raising concerns about the microperfusion performance (Figure 3A). Computational fluid dynamic simulations indicated, however, that even though the embryos rested directly on the inlets of suction channels, their rectangular cross sections still permitted for a considerable flow passing around the embryos (Figure 3B). These results on mass transfer inside the device were next validated experimentally using 0.04% Trypan Blue dye as a model probe while the perfusion flow rate was set to 0.4 ml/min. Figure 3C depicts both the simulated and real-world assessments of the mass transfer across the array fully loaded with a population of 48 zebrafish embryos. We found that dye freely entered all occupied traps and the complete dye exchange across the whole device occurred within 90 s (Figure 3C and D, Figure S2). This could be further accelerated to below 15 s simply by increasing the flow rate up to 2 ml/min (Figure 3D). Next we validated the dye delivery to each embryo across by perfusing the chip with a solution of 0.04% Trypan Blue and subsequently quantifying the intensity of the stained embryos (Figure 3E and F) after a washing step with E3 medium. This revealed uniform labeling with occasional higher intensities detected due to the heterogeneous sizes within the embryo population (Figure 3E and F). The above experiments were also performed using tetramethylrhodamine methyl ester (TMRM) fluorescent probe yielding comparable results. Our data demonstrated a strong correlation with the computational models that guided the design of the device providing further evidence that hydrodynamic trapping principles allow for both robust immobilization of the fish embryos inside the traps and also efficient medium exchange and uniform drug delivery across the miniaturized array.

The ability to perform continuous perfusion experiments without displacing the embryos is an important consideration in environmental toxicology in particular. Static tests performed traditionally in Petri dishes represent the simplest and cheapest way to assess toxicity of chemicals [7]. They can be, however, inadequate to test toxicity of many compounds because of their adsorption, degradation, metabolic inactivation, shortage of oxygen, uncontrolled changes in medium pH. All these condition can severely constrain the appropriateness of static exposure utilized in standard FET tests [7]. Therefore, it has been recently postulated by Lammer et al that flow-through/dynamic acute tests should become a preferable choice for toxicants analysis [7]. Furthermore as suggested by Lammer et al yet another drawback of static FET is that these use only very small volumes of test substance which make chemical confirmatory analyses tremendously difficult [7]. By implementing a perfusion system, larger volumes of medium can be collected for confirmatory chemical analysis such as the metabolic degradation of compounds. This is expected to bring greater quality control and data for a comprehensive interpretation of toxicity results [7].

Moreover, the device features the ability to pulse the embryos with the drug followed by the rapid medium exchange without disturbing the embryo position. This can be of particular importance to precisely control the transcriptional activation of target genes using inducible transgenes such as Tet-On Tetracycline-Inducible Gene Expression System. Moreover, the pulsed drug delivery can be exploited for the temporary delivery of toxins and/or anaesthetic agents to evaluate short-term exposures to high dosages, followed by the embryo development in drug free medium. Also the delivery of cell permeable fluorescent probes can be greatly facilitated by their quick wash out to reduce the fluorescent background during subsequent image acquisition.

### Microenvironment for Long-term Embryo Culture

**Figure 4:**
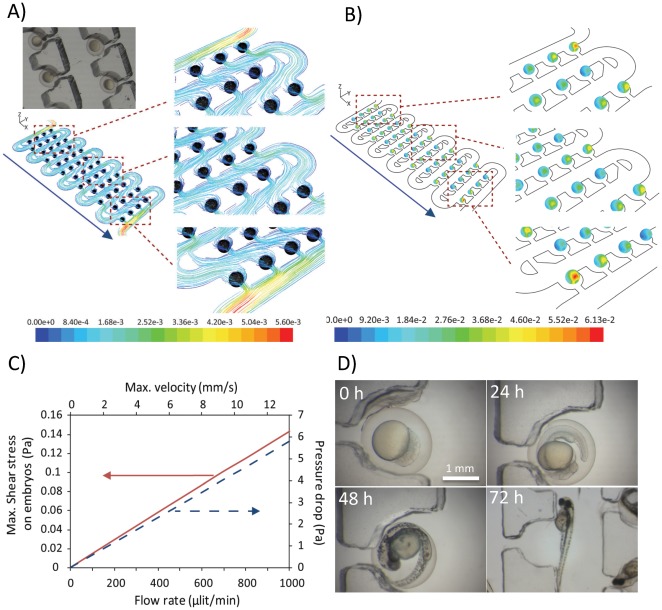
Assessment of microenvironmental conditions inside the chip: **A**) 3D streamline of flow obtained by computational fluid dynamic simulations across the fully loaded device. Perfusion was simulated at a volumetric flow rate of 0.4 ml/min; **B**) Contours of shear stress (Pa) exerted on embryos across the whole device. There sections of the chip (upper, middle and lower part) are magnified for clarity. Perfusion was simulated at a volumetric flow rate of 0.4 ml/min; **C**) Performance curves of the chip as a function of the volumetric flow rate; **D**) Time-lapse images of developing zebrafish embryos collected every 24 hours. Embryos were loaded on a chip at the volumetric flow rate of 2 ml/min. Subsequently the chip was perfused at a rate of 0.4 ml/min for up to 72 hours.

To demonstrate the feasibility for developmental analysis of zebrafish embryos over extended periods of time, we next performed numerical simulations to estimate both the flow velocity and the extent of shear stress exerted over embryos. Firstly, a 3D model of the entire device was built and analysis of flow profile obtained by computational fluid dynamic simulations across the fully loaded device. The embryos were simulated as rigid, not deformable spheres (Figure 4A). The distribution of shear stress inside the traps was then obtained from a 3D model (Figure 4B). The results indicated that the embryos encountered an average shear stress ranging from 1.83E–2 to 4.60E–2 Pa at the perfusion rate of 0.4 ml/min (Figure 4B and C). A maximum shear stress of 6.13E-2 Pa was to be experienced only by the embryos located in the first trap of each row (Figure 4B and C). This supported the notion that due to the nature of the flow inside the miniaturized trapping system the embryos will be kept within a low shear stress microenvironment. In this regard, we previously extensively explored signaling events associated with single cells under a range of flow induced mechanical loads [23], [30]. Our current results indicate that the trapped embryos generally experienced a shear stress at least two orders of magnitude lower than values reported to trigger cell signaling events [23], [30]. Moreover, in contrast to cells, embryos are protected by a robust physical barrier (chorion membrane) and therefore we anticipate that shear stress effects on developing embryos are negligible.

**Figure 5:**
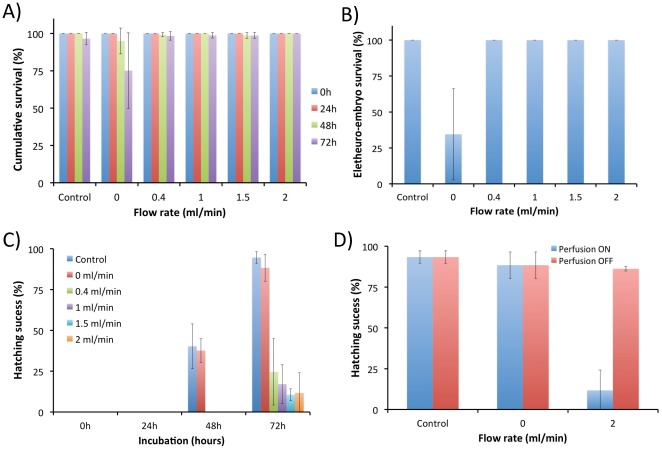
Development of zebrafish embryos on a chip: **A**) Cumulative survival (embryos and eletheuro-embryos) perfused on chip at varying volumetric flow rates. Control denotes static 60 mm Petri Dish; **B**) Survival of hatched eletheuro-embryos at 72 hours perfused on a chip at varying volumetric flow rates; **C**) Hatching success of eletheuro-embryos perfused on chip at varying volumetric flow rates. Control denotes static 60 mm Petri Dish; **D**) Hatching time and success of eletheuro-embryos perfused on chip can be dramatically improved when perfusion is disengaged at 72 hours. Control denotes static 60 mm Petri Dish.

Accordingly, we next validated these assumptions by performing a long-term culture of zebrafish embryos perfused on a chip at varying flow rates of 0.4 to 2 ml/min for up to 72 hours (Figure 4D, 3). We observed the normal and very uniform development of all embryos immobilized across the array (Figure 4D, Figure S3). Furthermore, during the standard ecotoxicological FET test period of up to 72 hours, we did not notice any discernible phenotypic effects irrespectively of the magnitude of flow rates. The cumulative survival of embryos and eletheuro-embryos cultured on chip for up to 72 hours was over 95% with the exception of a chip kept at a static regimen (Figure 5A). In the latter case, the high mortality amongst the hatched eletheuro-embryos was most likely due to the higher metabolic rate of hatched stages and oxygen deprivation when insufficient exchange of medium in the chip was present (Figure 5B). Interestingly, the hatching time and hatching success of eletheuro-embryos were inversely proportional to the volumetric flow rate (Figure 5C). We observed, however, that this could be dramatically improved when perfusion was disengaged at 72 hours (Figure 5D). Noticeably, the microperfusion culture did not slow down the embryo development process and our data indicate that following the chip disconnection, embryos immediately commenced the hatching process with up to 6.5 fold increase in number of hatched stages over only 2 hours (Figure 5D). This combined with the results from the static chip (where hatching success was comparable to the control 60 mm Petri Dish vessels) indicates that hydrodynamic immobilization rather than mechanical constriction inside the traps can somehow arrest the fish hatching process at the higher flow rates (Figure 5D). Based on our results, we postulate that our design is particularly suitable for the bioassay test period of up to 48–72 hours. When the hatching time and success is of importance, perfusion should be performed at much lower rates that enable widespread embryo hatching while preserving sufficient medium exchange to support survival of the eletheuro-embryo stages. Further studies are required to rule out any undetected and long-term effects that can become visible following recovery of juvenile stages.

Indeed, the chip design offered the capability to recover both embryos and swimming eletheuro-embryo stages. The recovery was obtained using a reversed flow leading to the hatched stages being collected from the inlet port (Figure S3). Otherwise the hydrodynamic forces overcame the swimming behavior and attracted eletheuro-embryos back to the trapping region (Figure S3). No mechanical damage to the recovered embryos was observed.

### On-chip Zebrafish in vivo Angiogenesis Assay

Zebrafish has recently emerged as an innovative, whole animal model for accelerated screening of small molecule drugs that affect blood vessel formation (angiogenesis) [21], [31], [32]. The optical transparency of embryos allows for convenient microscopic visualization of characteristic patterns of intersegmental vessels (ISV). This can be reportedly used as a surrogate bioassay to perform primary screens of investigational anti-angiogenic compounds that diffuse into the embryo and induce dose dependent inhibition of ISV formation [21], [31], [32].

**Figure 6:**
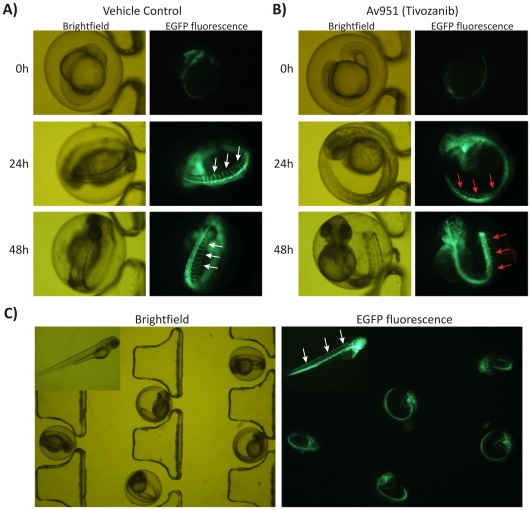
On-chip angiogenesis assay using transgenic zebrafish line: **A**) The transgenic fli1a:EGFP embryos at 16 hpf were loaded, immobilized and continuously perfused on a chip with E3 media containing vehicle control (DMSO); **B**) The transgenic fli1a:EGFP embryos at 16 hpf were loaded, immobilized and continuously perfused on a chip with a 1 µM of selective VEGFR inhibitor AV951 (Tivozanib, AVEO Pharmaceuticals Inc). Fluorescent and brightfield images were acquired at 0, 24 and 48 hours intervals. The optical transparency of embryos coupled with hydrodynamic immobilization on a chip array allowed for convenient microscopic visualization of characteristic patterns of intersegmental vessels (ISV, white arrows) and their AV951-induced inhibition (red arrows); **C)** Fli1a:EGFP transgenic embryos arrayed and hydrodynamically immobilized on a chip-based device. Developing patterns of intersegmental vessels are clearly visible even at the low magnification. Inset shows high magnification of hatched fli1a:EGFP larva with fully developed pattern of vasculature (white arrows).

Following our initial experiments, we set on to validate the applicability and performance of the microfluidic embryo array technology technology for the analysis of anti-angiogenic compounds using transgenic zebrafish line (fli1a:EGFP)^y1^ [21], [27]. Fli1a:EGFP line expressing enhanced green fluorescent protein in the vasculature represents a rapid way to visualize development of ISV formation [21]. In the microperfusion on-chip analysis, the fli1a:EGFP embryos were loaded onto a chip at 16 hpf stage before angiogenic sprouting of intersegmental and head vessels had begun. The embryos were continuously perfused with E3 media containing 1 µM of selective VEGFR inhibitor AV951 (Tivozanib, AVEO Pharmaceuticals Inc) [27], [28], and images were acquired every at 0, 24 and 48 hours intervals. Tivozanib is a novel, selective, inhibitor targeting of all three vascular endothelial growth factor (VEGF) receptors 1, 2 and 3 [28]. It has been designed to maximize the VEGF inhibition with minimized off-target toxic effects. Figure 6 presents representative images of single embryos entrapped on a chip and visualized using fluorescent stereomicroscopy for AV951-induced inhibition of ISV formation.

We demonstrated that it is possible to automatically load transgenic zebrafish embryos into the microfluidic device and continuously perfuse them with media containing anti-angiogenic drug (Figure 6). Complete inhibition of ISV was achieved during close-loop microperfusion on chip as shown in Figure 6. The non-stimulated (control) fli1a:EGFP embryos developed normal vasculature as evidenced by the presence of characteristic patterns of intersegmental vessels (Figure 6). The data shows that the novel quinolone-urea derivative and selective VEGFR inhibitor AV-951 (Tivozanib, AVEO Pharmaceuticals Inc) is a very effective inhibitor of angiogenesis (Figure 6) [28].

The optical transparency and embryo immobilization allowed for convenient ISV imaging without the need for re-focusing and specimen re-positioning. Address designation to each embryo during analysis substantially accelerated the data acquisition in contrast to conventional Petri dish assays. Moreover, the drug exchange and rapid delivery of the anesthetic Tricaine are performed automatically within seconds without embryo dislodgment or need for repetitive pipetting. This allows for prospective automation at a large scale. We postulate that the microfluidic embryo arrays could form the basis of an automated in vivo assay for anti-angiogenic drug screening routines. When coupled with intelligent pattern recognition algorithms, fluorescent ISV signals in immobilized transgenic embryos could be automatically quantifiable in a high-throughput fashion [33]–[35].

## Discussion

Automated and high-throughput assays on zebrafish embryos are still largely unavailable [1]. Despite some emerging progress in cytometric large particle analysis and robotic liquid handling, the embryo dispensing and treatment are mainly performed manually and bioassays carried out under sub-optimal, static microtiter plate conditions. None of currently available technologies allow for a an automated positioning, trapping, perfusion treatment and long-term immobilization of large numbers of single embryos for real-time developmental analysis. The manual sorting, acquiring and dispensing of embryos using conventional liquid handling procedures is very cumbersome, time consuming and error prone, limiting reproducibility and research productivity. There is, thus, a great need to develop innovative integrated technologies for automated loading, transport, positioning and long-term immobilization of large, millimeter scale embryos in ecotoxicology, drug discovery and reproductive medicine [1], [7], [9].

We therefore envisage that embryo sorting, capture, culture and analysis in microfluidic system, where the most tasks are performed automatically without disturbing the embryo, and without sudden changes to embryo environment, will prove to be better than conventional static culture. Recent noteworthy reports by Wielhouwer et al [18] and Yang et al [19] have showed that fish embryos can develop in a confined microfluidic environment and that Lab-on-a-Chip devices hold a substantial promise for miniaturized toxicological analysis. Both studies fell short, however, of providing the integrated and automated loading, positioning, long-term immobilization of large number of single zebrafish embryos [1], [9]. Accordingly, we have for the first time described an innovative miniaturized chip-based embryo array that provides address designation to each embryo during analysis, highly controllable fluidic microenvironment and post-analysis eleuthero-embryo stage recovery. Our innovative technology hits on a key point associated with the difficulties associated with manual, slow and non-reproducible laboratory operations during embryo manipulations and analysis. No such technology for embryo/organism-based screening exists at the moment.

In contrast to any previously described technologies, our device creates a dynamic embryo arrays that allows to: (i) transport embryos, (ii) immobilize them for convenient imaging, (iii) continuously deliver reagents and drugs under perfusion while under continuous real-time observation, and also (iv) retrieve specimens post-analysis for further processing. The trap-and-release integrated microfluidic system is designed for one-step and automatic loading, perfusion, analysis and recovery of specimens all in a single, monolithic integrated device with no moving parts. Importantly, the design achieves one-embryo-in-one-trap for convenient address designation to each embryo and the characteristics of the flow allow retrieving a trapped specimen from the array by displacing it back into the main channel and collecting at the inlet. Moreover, this design greatly facilitates: (i) staining or treatment without displacing the embryos; (ii) highly controllable fluidic microenvironment for analysis under continuous perfusion; (iii) spatial segregation of developing embryos to avoid embryo-to-embryo interaction; (iv) applicability of customized image and data analysis software, allowing address designation to each embryo.

Hydrodynamic techniques have been reportedly used to manipulate and trap single cells with diameters up to 50 µm [23], [29], [36]. Our work establishes new paradigm that that the underlying hydrodynamic principles can be also employed for manipulation of large particles with diameter well above 1 mm and mass often exceeding 1 mg. Notably, we have also simplified the fabrication process by combining high-speed laser prototyping with replica moulding in PDMS instead of conventional photolithography techniques. This has facilitated rapid design optimization and is scalable for a medium scale manufacturing of miniaturized devices for manipulation of fish embryos.

In closing, our work provides a new rationale for rapid and automated manipulation of developing zebrafish embryos inside the new class of miniaturized devices. We envisage that such technologies, where most tasks are performed automatically without disturbing the embryo, and without sudden changes to the embryo environment, will prove to be better and significantly more productive than conventional static and manual bioassays.

## Supporting Information

Figure S1 **Principles and validation of embryo trapping efficiency:** **A**) A 3D computer simulation of the flow velocity across each trap when device is perfused at a flow rate of 0.4 ml/min. Due to the computational limitations only first six rows were simulated. Due to mass continuity the flow velocity is highest across the first trap of each row. This support efficient trapping at a higher embryo velocities encountered in these regions due to the serpentine shape of the device; **B**) Pressure drop across each trap (Pa) obtained by numerical simulations. Analysis was performed at a simulated flow rate of 0.4 ml/min. Due to the computational limitations only first six row were simulated; **C–D**) Photographs of stages used to perform trapping efficiency experiments at the 22.5 and 45 degrees tilted angle respectively.
(TIFF)

Figure S2 **Validation of microperfusion and drug delivery inside the chip.** **A**) Mass transfer across the whole device (12 rows) fully loaded with zebrafish embryos. Chip was perfused with a 0.04% Trypan Blue dye at a volumetric flow rate of 0.4 ml/min. Red section denotes the first 6 rows simulated in Figure 3; **B**) Mass transfer across the simulated (left panel) and real-world (right panel) mesofluidic array without zebrafish embryos. Chip was perfused with a 0.04% Trypan Blue dye at a volumetric flow rate of 0.4 ml/min. Red section denotes the first 6 rows simulated in Figure 3; **C)** Comparative analysis of the mass transfer in chips with and without loaded embryos. Chip was perfused with a 0.04% Trypan Blue dye at a volumetric flow rate of 0.4 ml/min. Due to the computational limitations only first six rows were simulated.
(TIFF)

Figure S3 **Assessment of embryo development inside the chip.** **A**) Time-lapse images of developing zebrafish embryos collected every 24 hours. Embryos were loaded on a chip at the volumetric flow rate of 2 ml/min. Subsequently the chip was perfused at a rate of 0.4 ml/min for up to 72 hours. Only six rows are shown due to the limitation of the imaging stereoscopic system. Note the normal and very uniform development of embryos hydrodynamically immobilized on the microfluidic array; **B–D**) Microphotographs of hatched eletheuro-embryos at 72+ hours on a chip. Note that chip design offers the capability to recover both embryos and also swimming eletheuro-embryo stages. The recovery is best performed at the reversed flow rate when hatched stages can be collected from the inlet port. Otherwise the hydrodynamic forces will overcome the swimming behaviour and attract eletheuro-embryos back to the trapping region as denoted in D and E.
(TIFF)

Movie S1 **One step loading, hydrodynanic trapping and long-term immobilization of living zebrafish embryos on a miniaturized chip-based device.** Embryos were counter stained with 0.04% Trypan Blue to improve their visibility during the video-microscopy. Perfusion was conducted at a volumetric flow rate of 1 ml/min.
(MP4)
