# Supplementary figures and images for: Miniaturized Embryo Array for Automated Trapping, Immobilization and Microperfusion of Zebrafish Embryos
Source: PLoS One. 2012 May 14;7(5):e36630. doi: 10.1371/journal.pone.0036630 (PMC3351474; doi:10.1371/journal.pone.0036630)

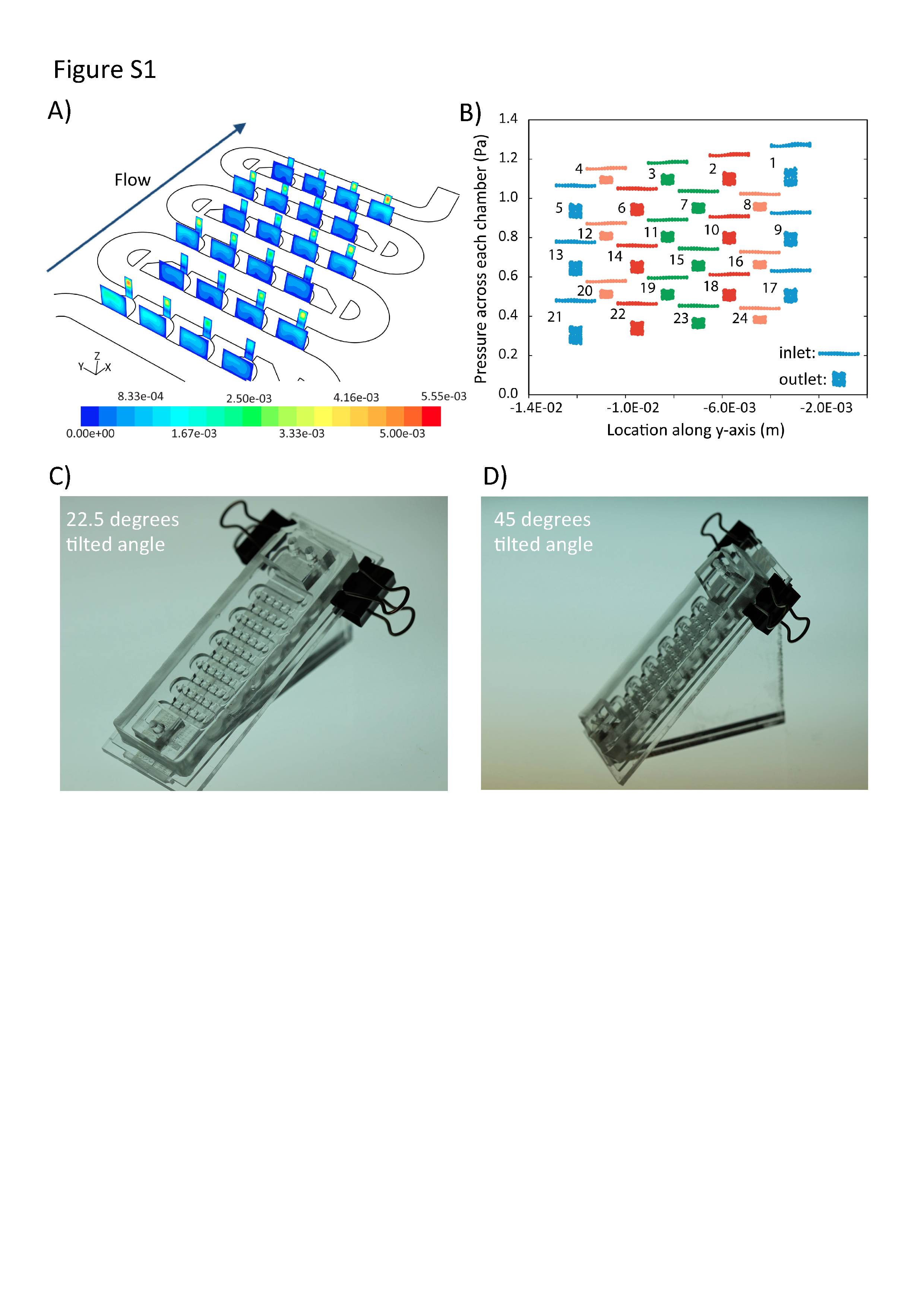

Supplement: Figure S1 — Principles and validation of embryo trapping efficiency: A) A 3D computer simulation of the flow velocity across each trap when device is perfused at a flow rate of 0.4 ml/min. Due to the computational limitations only first six rows were simulated. Due to mass continuity the flow velocity is highest across the first trap of each row. This support efficient trapping at a higher embryo velocities encountered in these regions due to the serpentine shape of the device; B) Pressure drop across each trap (Pa) obtained by numerical simulations. Analysis was performed at a simulated flow rate of 0.4 ml/min. Due to the computational limitations only first six row were simulated; C–D) Photographs of stages used to perform trapping efficiency experiments at the 22.5 and 45 degrees tilted angle respectively. (TIFF) [file pone.0036630.s001.tiff]

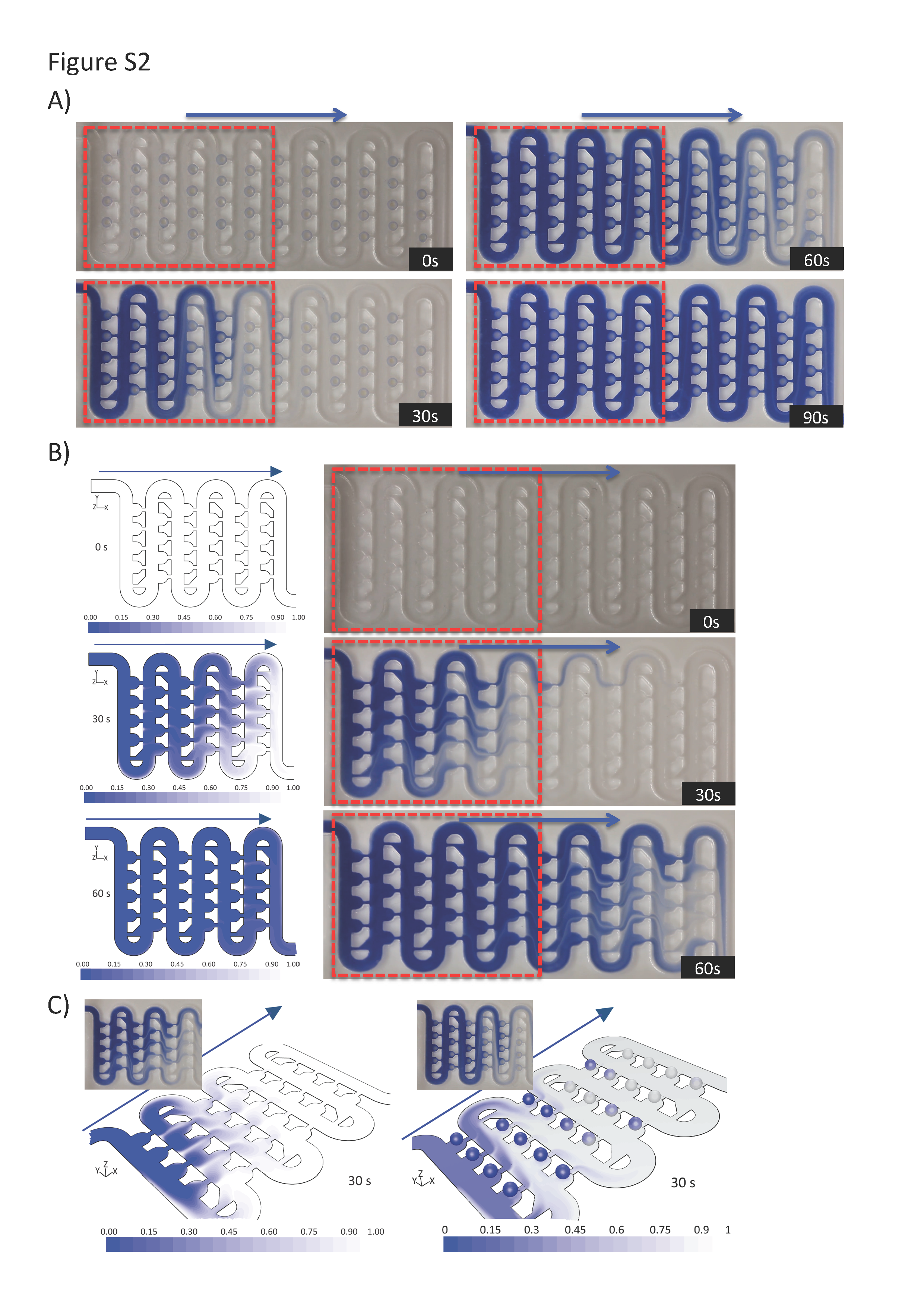

Supplement: Figure S2 — Validation of microperfusion and drug delivery inside the chip. A) Mass transfer across the whole device (12 rows) fully loaded with zebrafish embryos. Chip was perfused with a 0.04% Trypan Blue dye at a volumetric flow rate of 0.4 ml/min. Red section denotes the first 6 rows simulated in Figure 3; B) Mass transfer across the simulated (left panel) and real-world (right panel) mesofluidic array without zebrafish embryos. Chip was perfused with a 0.04% Trypan Blue dye at a volumetric flow rate of 0.4 ml/min. Red section denotes the first 6 rows simulated in Figure 3; C) Comparative analysis of the mass transfer in chips with and without loaded embryos. Chip was perfused with a 0.04% Trypan Blue dye at a volumetric flow rate of 0.4 ml/min. Due to the computational limitations only first six rows were simulated. (TIFF) [file pone.0036630.s002.tiff]

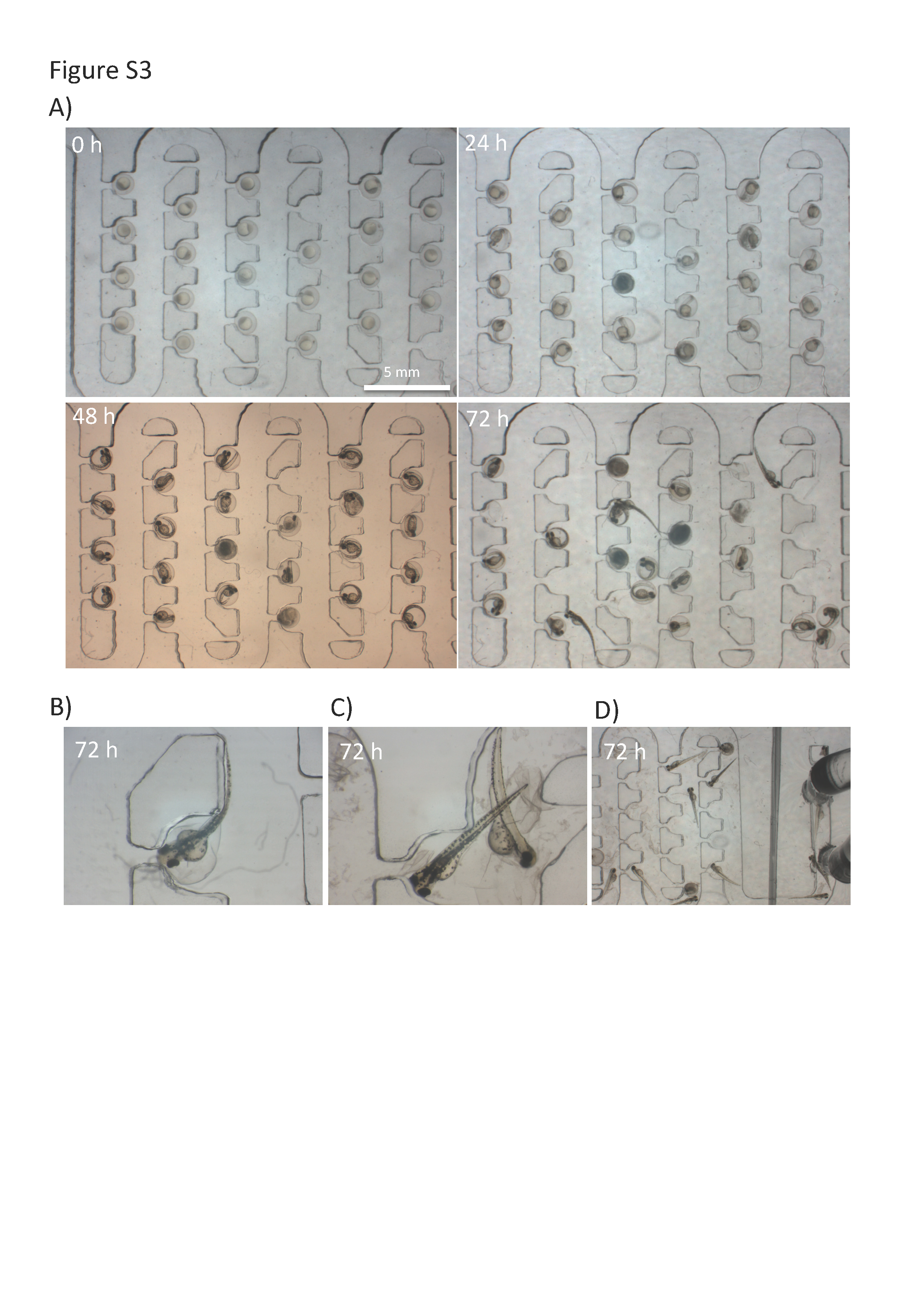

Supplement: Figure S3 — Assessment of embryo development inside the chip. A) Time-lapse images of developing zebrafish embryos collected every 24 hours. Embryos were loaded on a chip at the volumetric flow rate of 2 ml/min. Subsequently the chip was perfused at a rate of 0.4 ml/min for up to 72 hours. Only six rows are shown due to the limitation of the imaging stereoscopic system. Note the normal and very uniform development of embryos hydrodynamically immobilized on the microfluidic array; B–D) Microphotographs of hatched eletheuro-embryos at 72+ hours on a chip. Note that chip design offers the capability to recover both embryos and also swimming eletheuro-embryo stages. The recovery is best performed at the reversed flow rate when hatched stages can be collected from the inlet port. Otherwise the hydrodynamic forces will overcome the swimming behaviour and attract eletheuro-embryos back to the trapping region as denoted in D and E. (TIFF) [file pone.0036630.s003.tiff]
